# Astrocytes of the optic nerve exhibit a region-specific and temporally distinct response to elevated intraocular pressure

**DOI:** 10.1186/s13024-023-00658-9

**Published:** 2023-09-27

**Authors:** Arpan G. Mazumder, Amélie M. Julé, Daniel Sun

**Affiliations:** 1 Department of Ophthalmology, Schepens Eye Research Institute of Massachusetts Eye and Ear, Harvard Medical School, Boston, MA 02114 USA; 2 Department of Biostatistics, Harvard T.H. Chan School of Public Health, Boston, MA 02115 USA

**Keywords:** Optic nerve head, Optic nerve astrocytes, Glaucoma, Ribotag, Reactive astrocytes, Optic nerve transcriptional profile

## Abstract

**Background:**

The optic nerve is an important tissue in glaucoma and the unmyelinated nerve head region remains an important site of many early neurodegenerative changes. In both humans and mice, astrocytes constitute the major glial cell type in the region, and in glaucoma they become reactive, influencing the optic nerve head (ONH) microenvironment and disease outcome. Despite recognizing their importance in the progression of the disease, the reactive response of optic nerve head astrocytes remains poorly understood.

**Methods:**

To determine the global reactive response of ONH astrocytes in glaucoma we studied their transcriptional response to an elevation in IOP induced by the microbead occlusion model. To specifically isolate astrocyte mRNA in vivo from complex tissues, we used the ribotag method to genetically tag ribosomes in astrocytes, restricting analysis to astrocytes and enabling purification of astrocyte-associated mRNA throughout the entire cell, including the fine processes, for bulk RNA-sequencing. We also assessed the response of astrocytes in the more distal myelinated optic nerve proper (ONP) as glaucomatous changes manifest differently between the two regions.

**Results:**

Astrocytes of the optic nerve exhibited a region-specific and temporally distinct response. Surprisingly, ONH astrocytes showed very few early transcriptional changes and ONP astrocytes demonstrated substantially larger changes over the course of the experimental period. Energy metabolism, particularly oxidative phosphorylation and mitochondrial protein translation emerged as highly upregulated processes in both ONH and ONP astrocytes, with the former showing additional upregulation in antioxidative capacity and proteolysis. Interestingly, optic nerve astrocytes demonstrated a limited neuroinflammatory response, even when challenged with a more severe elevation in IOP. Lastly, there were a greater number of downregulated processes in both astrocyte populations compared to upregulated processes.

**Conclusion:**

Our findings demonstrate an essential role for energy metabolism in the response of optic nerve astrocytes to elevated IOP, and contrary to expectations, neuroinflammation had a limited overall role. The transcriptional response profile is supportive of the notion that optic nerve astrocytes have a beneficial role in glaucoma. These previously uncharacterized transcriptional response of optic nerve astrocytes to injury reveal their functional diversity and a greater heterogeneity than previously appreciated.

**Supplementary Information:**

The online version contains supplementary material available at 10.1186/s13024-023-00658-9.

## Introduction

Glaucoma is a complex disease characterized by the progressive dysfunction and irreversible loss of retinal ganglion cells (RGCs). It is one of the most common neurodegenerative diseases and the leading cause of irreversible blindness, affecting ~ 80 million people worldwide [1]. Elevated intraocular pressure (IOP) is a major risk factor and to date the only clinically available therapies target the reduction of IOP [2, 3]. Given the incomplete efficacy of these treatments, which often slow rather than halt progression [2, 4–6], there is a need to better understand the pathophysiology of the disease.

The optic nerve is an important tissue in glaucoma and many of the early neurodegenerative processes associated with glaucoma take place primarily in the unmyelinated optic nerve head (ONH) region, including changes in extracellular matrix composition, tissue remodeling, dysfunctional axonal transport, neuroinflammation, monocyte infiltration and axonal damage [7–12]. This is consistent with prior studies indicating that the characteristic pattern of ganglion cell loss in glaucomatous retinas is best explained by initial damage to axon bundles in the ONH region [13]. Astrocytes at the ONH are of particular interest in glaucoma because they are the predominant glial cell type in this region in both mouse and human [14–16]. As in other parts of the CNS, ONH astrocytes become reactive in injury and disease (including glaucoma), leading to changes in their morphology, function, and gene expression that can profoundly influence the ONH microenvironment and outcome [17–21]. Despite clear evidence of ONH astrocyte reactivity in experimental models of glaucoma [17–21] and tissue from glaucomatous donors [22], the reactive response of ONH astrocytes remains poorly understood.

Gene expression studies have been performed on whole ONH tissues, but this precludes clear attribution of transcriptional signatures to local astrocytes, even though they make up a large proportion of the ONH. This becomes particularly challenging when experimental glaucoma is induced, and differential gene expression is examined, as early monocyte infiltration significantly contributes to a pro-inflammatory environment of the ONH [23, 24]. For example, resident microglia show few changes in inflammatory molecules, but are metabolically active, a shift that is typically anti-inflammatory [24]; the net effect of changes in these cell types and those of the local vasculature is to confound efforts to isolate the changes undergone by ONH astrocytes. Others have utilized in vitro ONH astrocyte cultures to eliminate contributions from other cell types [25–28], however, astrocytes generally lose many of their distinct expression patterns in culture [29, 30]. Finally, while the ONH region is amenable to examination by immunolabeling with astrocyte markers, this approach is low throughput and ill-suited to studying the global astrocytic response to glaucoma.

To determine the overall reactive response of ONH astrocytes in glaucoma we studied their transcriptional response to a chronic elevation in IOP induced by a microbead injection. To specifically isolate astrocyte mRNA in vivo from complex tissues, we used the ribotag method to genetically tag ribosomes in astrocytes, restricting analysis to astrocytes and enabling purification of astrocyte-associated mRNA throughout the entire cell, including the fine processes, for bulk RNA-sequencing [31–34]. We also assessed the response of astrocytes in the more distal myelinated optic nerve proper (ONP) as glaucomatous changes manifests differently between the two regions. Our results demonstrate that ONH and ONP astrocytes each have distinct temporal and transcriptional responses to elevated IOP. Energy metabolism, particularly oxidative phosphorylation and protein metabolism were key upregulated processes, while neuroinflammation was limited, even with a more severe elevation in IOP. These previously uncharacterized transcriptional responses reveal their functional diversity to injury and a greater heterogeneity than previously appreciated.

## Methods

All animals were handled in accordance with the ARVO Statement for the Use of Animals in Ophthalmic and Vision Research, and all procedures were approved by the Institutional Animal Care and Use Committee at Schepens Eye Research Institute.

### Animals

Mice aged 3–4 months old were housed in a temperature and humidity-controlled facility with 12 h light/dark cycle and received food and water ad libitum. The study used two strains: (1) B6J.129(Cg)-Rpl22^tm1.1Psam^/SjJ (The Jackson Laboratory, Bar Harbor, ME, #029977), also known as ribotag mice, and (2) B6.Cg-Tg (GFAP-Cre) 73.12Mvs/J mice (The Jackson Laboratory, Bar Harbor, ME, #012886). The ribotag method allows efficient isolation of ribosome-associated mRNA transcripts from complex tissues in a cell-type specific manner [31–33]. The ribotag mouse has a modification in the 60S ribosomal protein gene, Rpl22, such that in the presence of Cre recombinase the floxed exon 4 of Rpl22 will be replaced with a hemagglutinin (HA)-tagged exon 4, effectively labeling the cells’ polyribosomes so that they can be isolated by immunoprecipitation. Ribotag mice can be crossed to a variety of Cre recombinase driver mouse lines, and in our case, we use a constitutive GFAP-Cre line in which a mouse GFAP promoter sequence directs expression of Cre recombinase in astrocytes. Both mice lines were on a C57Bl/6 background. We initially crossed GFAP-Cre recombinase expressing mice with hemizygous ribotag mice to get offspring heterozygous for the ribotag allele (Rpl22^HA/+^). Heterozygous ribotag mice were then bred together to obtain experimental mice that were homozygous for the ribotag allele with the Cre transgene (Rpl22^HA/HA^Cre^+^).

### Microbead model

We used the microbead occlusion model to elevate IOP in one eye. The microbeads are inert and have been used in numerous studies of experimental glaucoma [17, 20, 33, 35–37]. A glass microneedle was introduced through a corneal puncture initially created by a 30.5-gauge needle and 2 µl of 15 µm diameter polystyrene microbeads (Thermo Fisher Scientific; #F8841) was injected (final concentration of 2.7 × 10^7^ beads/ml suspended in saline). This method induces an elevated IOP that lasts 4 weeks and peaks at 23–27 mmHg at 7–10 days post-injection. In some animals, a more severe elevation in IOP was induced by injecting 3.5 µl of the microbeads (higher elevation in IOP; final concentration of 4.01 × 10^7^ beads/ml) elevating the IOP to 30–33 mmHg at 7 days. The contralateral eye was untreated. A separate control group of ribotag mice received a saline injection in-lieu of the microbeads and they demonstrated normal IOPs over the 30 day experimental period. Mice were regularly examined on a slit lamp for signs of any inflammatory response or overt damage in the anterior segment. Mice that showed any of these signs were excluded.

### Measuring IOP

Mice were anaesthetized by isoflurane (2–4%; Webster Veterinary) delivered in 100% oxygen via a precision vaporizer. Measurements were taken 4–5 min after animals lost consciousness, which was defined as failure to detect motion in response to forced movement and absence of eye blinking. The IOPs were measured in both eyes 1 d before microbead injection and then every 3 d afterward using a tonometer (TONOLAB; Icare). Measurements were made at the same time in the morning to account for diurnal variations. The tonometer takes five measurements and based on this gives a single mean. We considered this as one measurement; five measurements were made from each eye and the mean was calculated to represent the IOP. IOPs were measured by individuals blinded to the genotype of the animal and the injury induced.

### Preparation of reagents for ribotag immunoprecipitation

#### Homogenization buffer (HB)

The homogenization buffer was prepared by adding 2.5 mL of 2 M KCl (RNase free; Fisher Scientific, #AM9640G), 2.5 mL of 1 M Tris (pH 7.4; ThermoFisher Scientific, #17,926), 500 µl of NP-40 (ThermoFisher Scientific, #28,324) and 600 µl of 1 M MgCl2 (RNase free; Fisher Scientific, #AM9530G) into a 50 mL Falcon tube. The volume was adjusted to 50 mL with DNase/RNase free water and vortexed until all contents were completely dissolved. The supplemented homogenization buffer (HB-S) was always prepared fresh prior to homogenization by adding 50 µl of protease inhibitor (VWR, #97,063–970), 5 µl of 1 M DTT (VWR, #97,061–340), 25 µl of RNasin (VWR, #PAN2615), 50 µl of Heparin (100 mg/ml; Fisher Scientific, #BP252420) and 100 µl of cycloheximide (5 mg/ml; Fisher Scientific, #AC357420050) in a 15 ml Falcon tube. Finally, the volume was adjusted to 5 mL with HB buffer.

#### High salt buffer (HSB)

To prepare the high salt buffer, 7.5 mL of 2 M KCl (RNase free; Fisher Scientific, #AM9640G), 2.5 mL of 1 M Tris (pH 7.4; ThermoFisher Scientific, #17,926), 600 µl of 1 M MgCl_2_ (Rnase free; Fisher Scientific, #AM9530G), and 500 µl of NP-40 (ThermoFisher Scientific, #28,324) were added in a 50 mL Falcon tube. The final volume was made up to 50 mL with DNase/RNase free water and vortexed to completely dissolve all contents. The high salt buffer supplemented (HSB-S) was prepared fresh prior to the washing step by adding together 10 µL of protease inhibitor (VWR, #97,063–970), 40 µl of cycloheximide (5 mg/ml; Fisher Scientific, #AC357420050) and 5 µL of RNasin (VWR, #PAN2615) in a 2 mL Eppendorf tube and the volume was adjusted to 2 mL with HSB.

### Preparation of tissue and ribotag immunoprecipitation

The ribotag immunoprecipitation technique has been optimized from Sanz et al., [31, 32] for very small tissues and previously published by us [33]. Euthanasia was performed with carbon dioxide followed by cervical dislocation. The skull and the brain were removed and each eye together with the optic nerve carefully dissected out, being careful to minimize mechanical stress or damage to the tissue. The ONH and ONP were micro-dissected in chilled PBS on ice. We considered the translucent unmyelinated portion of the optic nerve as the ONH. To obtain sufficient RNA, 4 ONHs and 4 ONP (2 males and 2 females) were pooled for *N* = 1 each. Each of the ONH and ONP came from the same mouse and an equal length of ONP as ONH was used. We used the most proximal portion of the ONP, and the remainder of the optic nerve was not sequenced. Once dissected, tissues were placed in 150 µl of HB-S buffer, centrifuged for 2 min at 1000 rpm at 4 °C, then homogenized using a PYREX Glass Pestle in the microcentrifuge tubes on ice. The lysate was further centrifuged for 10 min at 10,000 rpm at 4 °C, then the supernatant collected. 10 µl of the supernatant was mixed with 350 µl of RLT lysis buffer (RNeasy Micro Kit, Qiagen, #74,034) and kept as Input, the remaining supernatant was used for immunoprecipitation (IP).

HA-tagged ribosomes were captured by adding 2.5 µl of anti-HA antibody (BioLegend, #MMS-101R) to the IP supernatant and incubated at 4 °C for 4–5 h on a mini-tube rotator with gentle mixing. This antibody-supernatant mixture was then added to 55 µl of pre-washed Pierce Protein A/G magnetic beads (Thermo Fisher Scientific, #88,802) and incubated overnight at 4 °C on a mini-tube rotator. The following day, the Eppendorf tubes were placed in a Dynamag-2 magnetic rack on ice and washed twice with HSB-S buffer. The non-precipitated flow-through from each immunoprecipitated sample was collected and RNA was purified for enrichment analysis using a RNeasy Micro Kit (Qiagen, #74,034).

### RNA-seq library generation and sequencing

After RNA extraction the quantity and quality were assessed on an Agilent 2100 Bioanalyzer. Typical RIN values and RNA quantity are shown in Table S1. The cDNA libraries were made from 0.62–15.9 ng of RNA. To avoid batch effects, the cDNA library preparation and sequencing were done at the same time for all samples. mRNA extraction was performed with oligo-dT beads, capturing poly(A) tails, and cDNA library preparation was done with the Smart-Seq HT Ultra-low input RNA kit (Takara Biosciences, #634,437). The cDNA samples were normalized and sequenced on an Illumina HiSeq 4000 with paired end 2 × 150 bp reads at 20–30 million reads per sample. cDNA Library preparation, sequencing and quality control checks were performed by Genewiz (Burlington, MA).

### RNA-seq mapping, analysis, and statistics

The sequencing data was processed for quality control, alignment to the mouse genome and transcript quantification using the bcbio-nextgen bulk RNA-seq pipeline (version 1.2.8-1c563f1). More precisely, reads were aligned using the STAR aligner (version 2.6.1d) to NCBI build mm10 of the mouse genome (Mus musculus). Transcript-level information was obtained from Ensembl release 94. Sequencing data quality control was executed by FastQC (version 0.11.8), Samtools (version 1.9), Qualimap (version 2.2.2d) and summarized with MultiQC (version 1.9). Transcripts were quantified by a quasi-alignment approach using Salmon (version 0.14.2). Transcript-level counts were then aggregated at the gene level and introduced into R using tximport. Downstream analyses were performed in R (version 4.1.0). Independent, pairwise differential expression analyses comparing gene expression between two interest groups were accomplished with DESeq2 (version 1.34.0). For subsequent functional analyses and graphical representations, DESeq2-derived log2 fold change values were corrected using the ashr shrinkage estimator. Gene set enrichment analyses (GSEA) and over-representation analyses (ORA) were performed using clusterProfiler (version 4.2.2) and ReactomePA (version 1.38.0). The differential gene expression was considered significant if the adjusted *p*-value < 0.05, applying Benjamini-Hochberg’s procedure for multiple comparisons adjustment. No cutoff on log2 fold change was used to define significant genes.

### Transcription factor analysis

We applied motif analysis algorithms to identify transcription factors (TFs) that may drive the observed variations in the transcriptomic program of ONH and ONP astrocytes over time following injury. DoRothEA provides a database of known TF-target gene pairs (regulons) and a weight for each pair reflecting the level of confidence and directionality of the interaction (activation/inhibition). Starting from the normalized gene expression data, decoupleR applies a weighted mean approach to calculate an enrichment score in the targets of a TF, or “TF activation score”, reflecting whether the transcriptomic profile in a sample is representative of the transcriptome expected downstream of a given TF. The top-most differentially activated TFs between groups of interest (e.g., ONH astrocytes at control and different timepoints post-injury) can then be determined, and candidate TFs driving variations in reactive astrocyte profiles are selected for validation experiments (e.g., duplex in-situ hybridization and immunohistochemistry). In parallel, HOMER was used to identify enriched TF binding motifs with known binding affinities, and the MEME suite of tools for motif discovery and motif enrichment was applied to identify de novo binding motifs that were then compared to databases of TFs with known binding profiles to infer potential regulatory mechanisms. The region 2 kb upstream and downstream of a gene’s TSS was used for motif analysis.

### Immunohistochemistry

Optic nerve sections were incubated with blocking solution (0.5% Triton-X, 10% donkey serum, 1% BSA) for one hour, then with the primary antibodies for one hour at room temperature. Primary antibodies used were: HA (1:500; BioLegend), SOX9 (1:100; R&D Systems), IBA1 (1:400; Wako), OLIG2 (1:100; R&D Systems), NG2 (1:400; EMD Millipore), LCN2 (1:400; R&D Systems). They were then washed in PBS (pH 7.4; 3 × 5 min) and incubated with secondary antibodies for one hour at room temperature. Secondary antibodies used were: Alexa Fluor 488-Donkey Anti-Mouse IgG (1:800; Jackson ImmunoResearch Labs), Alexa Fluor 594-Donkey Anti-Goat IgG (1:800; Jackson ImmunoResearch Labs), Alexa Fluor 594-Donkey Anti-Rabbit IgG (1:800; Jackson ImmunoResearch Labs). The tissues were then washed again in PBS (pH 7.4; 3 × 5 min) and cover slipped with Prolong Diamond Antifade Mountant (Thermo Fisher Scientific, #P36970). Slides were imaged on a Leica TCS SP8 confocal microscope. All fluorescent images in the figures are maximum intensity projections. The contrast and brightness of the final images were adjusted by Adobe Photoshop 2023; no other digital image processing was performed.

### In-situ hybridization

In-situ hybridizations were carried out according to the manufacturer’s instructions using the RNAscope duplex detection chromogenic kit (Advanced Cell Diagnostics, #322,500). All the probes were purchased from Advanced Cell Diagnostics. Optic nerves were fixed overnight at 4 °C in 4% paraformaldehyde and cryoprotected in 30% sucrose in 1 × PBS. In-situ hybridization was performed on 15 µm thick tissue sections and hybridized with mouse GFAP (#313,211) and LCN2 (#313,971). To assess probe specificity, two types of controls were used: (1) mouse PPIB (peptidylprolyl isomerase B, #313,911) and mouse Polr2a (polymerase II polypeptide A, #312,471-C2) as positive controls, and (2) bacterial DapB (dihydrodipicolinate reductase, #310,043) as a negative control. Tissue sections were counterstained with 50% Mayer’s hematoxylin, mounted using Vecta Shield mounting medium (Vector Laboratories, #H-1500), and bright-field images were obtained with a Nikon Eclipse 800 microscope interfaced with Olympus DP controller software.

### Quantitative RT-PCR

RNA was extracted using the RNeasy Plus Micro Kit (Qiagen, #74,034) and the quality and quantity were assessed on an Agilent 2100 Bioanalyzer. cDNA synthesis was performed using the Smart-Seq HT kit (Takara Biosciences, #634,437) and quantified by the Qubit dsDNA HS Assay Kit (Thermo Fisher Scientific, #Q32851). RT-qPCR was performed on a StepOnePlus Real-time PCR system (Applied Biosystems) using the SYBR Green PCR Master Mix (Applied Biosystems, ThermoFisher Scientific, Waltham, MA). Primers targeted astrocytes, microglia, oligodendrocytes, NG2 glial cells and axons. Melting curves were examined to verify amplification specificity. Each gene and glyceraldehyde phosphate dehydrogenase (GAPDH) were interrogated in triplicates. All technical replicates had cycle thresholds (CT) that differed from each other by less than 1.0 CT and a CT standard deviation < 0.3. All gene expression levels were normalized to GAPDH using the ddCT method.

### Assessing ATP levels

The ATP level was determined using a colorimetric test kit (Sigma, #MAK190). Four ONHs (2 males and 2 females) were pooled for *N* = 1. Four control (*N* = 4) and 30 day microbead injected (*N* = 4) C57Bl/56 mice were used. Tissue samples were immediately homogenized with 60 µl of ATP assay buffer (provided in the kit). The homogenates were then centrifuged at 5000 rpm for 10 min at 4 °C. The supernatant was incubated at 37 °C for 30 min after the addition of the ATP probe in the presence of a developer, and the ATP level was quantified by measuring absorbance at 570 nm with a Synergy2 BioTek micro plate reader interfaced with Gen5 controller software.

### Assessing reactive oxygen species

Reactive oxygen species was measured using the indicator dihydroethdium (DHE, Thermo Fisher Scientific, #D1168) on live unfixed optic nerve sections. DHE is specific for superoxides and hydrogen peroxides. Following removal of the eye, it was immediately embedded in OCT unfixed and frozen at -80 °C until use. 16 µm sections were cut on a cryostat and incubated in Hoechst for 15 min at 4 °C. Sections were then incubated in 0.025 μM DHE solution for 30 min at 37 °C in the dark. Sections were then washed with 1 × PBS, cover slipped with Prolong Diamond Antifade Mountant (Thermo Fisher Scientific, #P36970) and immediately photographed using a Leica TCS SP8 confocal microscope. Sections from normal untreated and 30 days microbead injected mice were always processed and imaged together under identical conditions.

### Assessing mitochondrial biogenesis

Tissues were obtained as in the assessment of ATP levels. DNA was extracted using the DNeasy blood and tissue kit (Qiagen, #69,504). RT-qPCR studies were done to determine mitochondrial DNA copy number using the NovaQUANT Mouse Mitochondrial to Nuclear DNA Ratio Kit (Millipore Sigma, #71,621) according to the manufacturer's protocol. This kit includes plates pre-aliquoted with primers for comparing the levels of two nuclear genes (BECN1 and NEB) to two mitochondrial genes (trLEV and 12 s) in a qPCR reaction. Two nanograms of DNA samples were then mixed with the appropriate amount of SYBR Green Master Mix (Applied Biosystems, #4,368,708) and qPCR was performed in an Applied Biosystems StepOnePlus PCR system with the following cycling conditions: 95 °C for 10 min, followed by 40 cycles of 95 °C for 15 s, 60 °C for 1 min. The mitochondrial DNA copy number was determined using the relative copy number approach. To calculate mitochondrial DNA copy number, we determined *N* = 2^−ΔCt^ from both trLEV/BECN1 pair (ΔCt1) and the 12 s/NEB pair (ΔCt2), where ΔCt1 = Ct_trLEV1 – Ct_BECN1 and ΔCt2 = Ct_12s – Ct_NEB, then we took an average of 2^−ΔCt1^ and 2^−ΔCt2^.

### Assessing glycolysis and oxidative phosphorylation

Glycolysis and oxidative phophosrylation were measured using an assay from Dojindo (#G270) in accordance with the manufacturer's instructions. Samples were obtained as in the assessment of ATP levels, with the addition of 4 ONPs (2 males and 2 females for *N* = 1). ONHs and ONPs were taken from the same mouse and each ONP sample was dissected to be the same length as each ONH. 2.5 µM oligomycin solution in HBSS (ThermoFisher Scientific, #24,020,117) was prepared from a 10 mM oligomycin stock solution. Oligomycin is used to inhibit ATP synthesis by oxidative phosphorylation. A volume of 100 µl of HBSS solution supplemented with oligomycin, was added into specific wells of a 96-well plate and an untreated HBSS solution was used as a control for the test. ONH and ONP tissues were placed in their respective wells and homogenized as mush as possible until no large pieces were visible. The 96-well plate was then incubated for 1.5 h at 37 °C. After that, 100 µl of ATP solution (made according to kit guidelines) was added and incubated at 25 °C for 10 min. The luminescence values were then measured using a Synergy2 BioTek microplate reader connected to Gen5 controller software. Finally, the metabolic shift was evaluated by comparing the luminescence values (a measure of the quantity of ATP detected) of ONH and ONP samples with and without oligomycin treatment.

### Retinal ganglion cell counts

Images of whole mounted retinae were obtained as z stacks (0.50 µm step size) at a magnification of 1.0. Each retina was divided into four quadrants and images were taken at two mid-peripheral regions per quadrant, for a total of eight images per retina. Cells that colocalized both BRN3A, a ganglion cell–specific marker and the nuclear dye Hoechst were counted. Ganglion cell density per retina was calculated as the mean of the eight regions. The percentage of ganglion cell loss was calculated by comparing the ganglion cell density of the microbead injected eye to the untreated contralateral eye. All the cell counting was performed by individuals blinded to the genotype of the animal and the injury induced.

### Quantification and statistical analyses

Statistical details of experiments can be found in the figure legends. Statistical analysis of the RNA-seq data is described above. Immunohistochemistry and duplex in-situ hybridizations were performed on *N* = 5 biological replicates.

### Availability of data and materials

The dataset supporting the conclusions of this article have been deposited at GEO (GSE237073 microbead injected animals. GSE211515 normal untreated animals) and publicly available at the date of publication.

## Results

### Ribotag approach to assess optic nerve astrocyte-specific transcriptional changes following elevated intraocular pressure

**Fig. 1:**
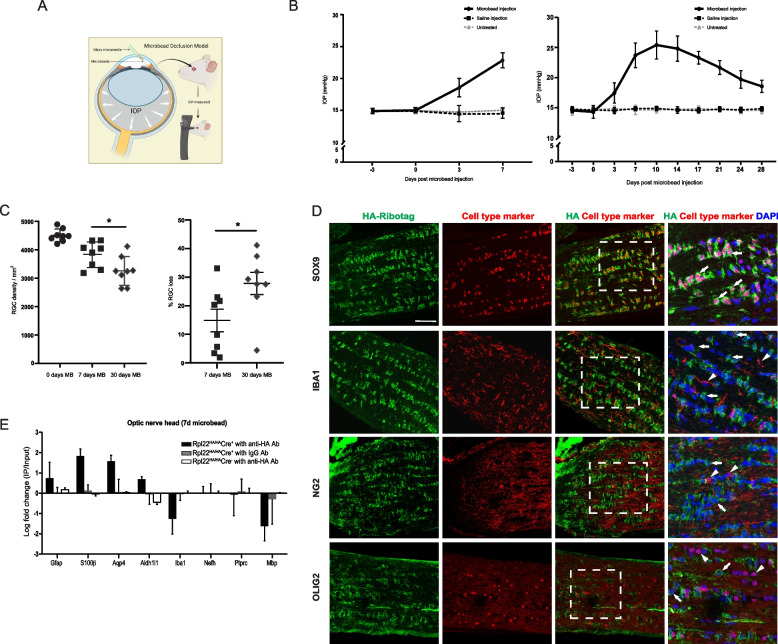
Characterization of the microbead occlusion model and the ribotag mouse. **A** Schematic depicting the microbead occlusion model of elevating the IOP followed by IOP measurement. **B** IOP measures for the two cohorts of animals that were sacrificed at 7 and 30 days. *N* = 6 for each of the 3 independent groups for each 7 and 30 days; microbead injected mice, saline injected mice, and naïve untreated mice. Mean ± SEM. **C** Loss of ganglion cell density in the treated eye and loss of ganglion cell in the treated eye as a percentage of the untreated contralateral eye. *N* = 8 biological replicates. * *p* < 0.05. Paired students t-test. MB = microbead injected. **D** Immunostaining for HA and cell-specific markers in the optic nerve of Rpl^HA/HA^/Cre^+^ mice. All images are longitudinal sections of the nerve head region, with the retinal end of the optic nerve on the left. Scale bar in top left panel = 50 µm and applies to all panels. **E** RT-qPCR of the immunoprecipitate (IP) and input from 7 day microbead injected optic nerve heads showing enrichment of astrocyte ribosomal mRNAs over other cell types. *N* = 4 per group. Mean ± SEM

Rpl22^HA/HA^Cre^+^ mice underwent unilateral microbead injections to elevate IOP (Fig. 1A**-**C) and mice were sacrificed at 7 and 30 days post-injection. We purified the astrocyte-specific ribosomal mRNA via immunoprecipitation using an antibody against HA. We previously showed that in the optic nerves of naïve Rpl22^HA/HA^Cre^+^ mice HA expression is specific for astrocytes and that the immunoprecipitated fraction is enriched for astrocyte markers and depleted for markers of microglia, oligodendrocytes, NG2 cells and axons [33]. Microbead injections did not change the specificity of HA expression, which colocalized with astrocyte marker SOX9 and not with markers for microglia (IBA1), oligodendrocytes (OLIG2), or NG2 glial cells (NG2) (Fig. 1D). Likewise, the immunoprecipitated fraction was only enriched for astrocyte markers and not for markers of other cell types (Fig. 1E).

### Cluster analysis reveals temporally distinct phases of the optic nerve head astrocyte response to elevated intraocular pressure

**Fig. 2:**
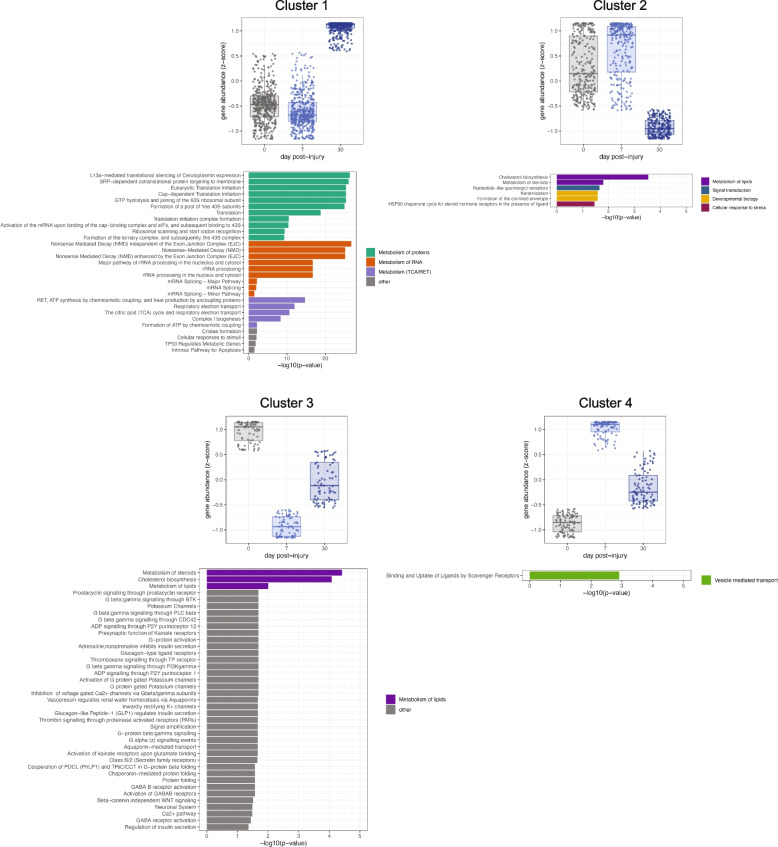
Cluster analysis reveals temporally distinct phases of the optic nerve head astrocyte response to elevated IOP. Cluster analysis using likelihood ratio test (LRT) on the 15,218 genes found to be differentially expressed across time and condition. This analysis determines sets of genes that exhibit similar variations in expression over time, and which can then be tested for pathway enrichment. 905 significantly variable genes were identified with an adjusted p-value cutoff of 0.05. Over-representation analysis, using the Reactome database was used to determine pathways (q-value < 0.2) enriched for genes from each cluster

We began our analysis of the effects of elevated IOP on ONH astrocytes by examining how global gene expression levels vary over time using a likelihood ratio test (LRT). This analysis determines sets of genes that exhibit similar variations in expression over time, and which can then be tested for pathway enrichment. We focused on the ONH region because of its importance as a site of neurodegeneration in glaucoma. A total of 15,218 genes were tested for differential expression across time in the microbead injection condition using the LRT model. Of these, 905 significantly variable genes were identified (adjusted *p*-value < 0.05) and could be grouped into four statistically unique clusters (Fig. 2 and see Table S2 for genes in each cluster): 1 (466 genes), 2 (266 genes), 3 (77 genes), and 4 (96 genes). The two largest gene clusters (Clusters 1 and 2) demonstrated a temporal pattern reflective of a late response in that there was no significant change between 0 and 7 days—surprising as this coincided with the period of IOP increase in our microbead model—followed by an increase (Cluster 1) or decrease (Cluster 2) towards 30 days, a period when the IOP gradually declined. Over-representation analysis (ORA) against the Reactome database determined Cluster 1 to be primarily enriched in pathways for protein and RNA metabolism (encompassing genes encoding for eukaryotic translation initiation factors and ribosomal proteins), and oxidative phosphorylation (respiratory electron transport, ATP synthesis, Complex I biogenesis, and TCA cycle). Here, most genes encoded for complexes of the respiratory electron transport chain, in particular complex I, the complex producing the most reactive oxygen species (Fig. 2; Table S2) [38]. Cluster 2 was particularly enriched for cholesterol biosynthesis, steroid metabolism, and keratinization and cornification.

In contrast, Clusters 3 and 4 showed an early response, with either a sharp decline (Cluster 3) or increase (Cluster 4) between 0 and 7 days, followed by a return towards baseline. Cluster 3 showed enrichment in the greatest number of pathways, however many contained very few genes and the same set of genes. The most distinguishing were the top 3, cholesterol biosynthesis, and steroid and lipid metabolism. Cluster 4 was enriched in a single pathway with only 4 associated genes (*Apol9b, Calr, Hsp90b1, Stab1*). For each of the 4 clusters, notable genes associated with glaucoma, neuroinflammation, or reactive astrocytes were: Cluster 1 (*Nrf2*, *Hspb1*, *S100β*), Cluster 2 (*Ptgs2*, *Clcf1*), and Cluster 4 (*C2*, *Tlr4*, *Lcn2*, *H2-T23*, *Thbs1*, *Il6ra*) (Table S2).

These results demonstrate important characteristics of the ONH astrocyte transcriptional response: (1) there were at least two temporally distinct phases of the response, (2) there was limited significant early response (e.g., 0 to 7 days); the majority of transcriptional changes happen later in injury (e.g., after 7 days, Clusters 1 and 2), (3) elevations in IOP primarily upregulate oxidative phosphorylation and RNA and protein metabolism, and downregulate cholesterol biosynthesis, and steroid and lipid metabolism, and (4) astrocytes showed a very limited neuroinflammatory response.

### Optic nerve head astrocytes upregulate oxidative phosphorylation, proteolysis and antioxidative capacity following elevated intraocular pressure

**Fig. 3:**
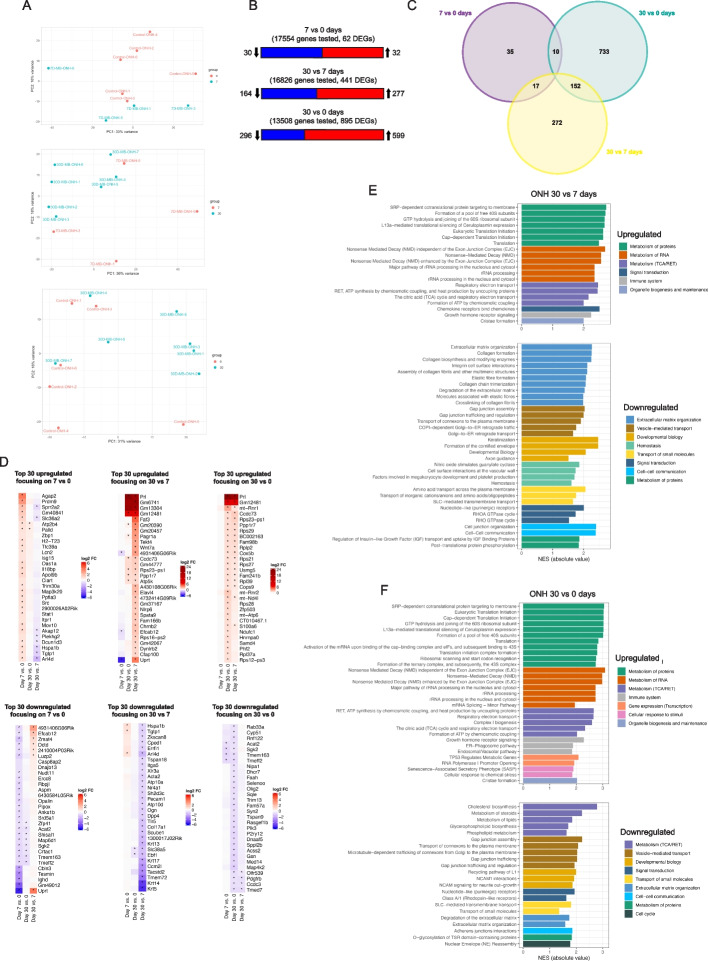
Transcriptional response of optic nerve head astrocytes to elevated IOP. **A** Principal component analysis based on expression levels of the top 2000 most variable genes across 7 vs 0, 30 vs 7 and 30 vs 0 day ONH samples. **B** Number of DEGs for each independent time point pairwise comparison. Significance was adjusted *p*-value < 0.05; no log2 fold change cutoff was applied. **C** Venn diagram showing the number of unique and overlapping DEGs across the 3 independent time point comparisons (adjusted *p*-value < 0.05). **D** Top 30 up and downregulated genes for each of the 3 independent time point comparisons, based on rank from largest to lowest log2 fold change value (absolute value, log2 FC > 0 upregulated, log2 FC < 0 downregulated). **E** Up and downregulated pathways from the 30 vs 7 day sample. Based on over-representation analysis of the up and down regulated genes using the Reactome database. Listed are all pathways with adjusted *p*-value < 0.05. Each pathway is color matched for the broader category it belongs to. NES refers to the normalized enrichment score that accounts for differences in gene set size and in correlations between gene sets and the expression dataset. **F** Up and downregulated pathways from the 30 vs 0 day sample. Based on over-representation analysis of the up and down regulated genes using the Reactome database. Listed are all pathways with adjusted *p*-value < 0.05. Each pathway is color matched for the broader category it belongs to

Having considered the global patterns of variations over time using the LRT analysis, we next examined whether a pairwise time point comparison would demonstrate previously unappreciated differences. We performed a pairwise comparison between time points, beginning with the ONH. The control data (0 days) we use here was generated in [33]. Examination of the 7 vs 0 (early) and 30 vs 7 day (late) datasets explored transcriptional processes during the injury period when IOP is rising and resolving, while the 30 vs 0 dataset explored changes that persisted at 30 days. Principal component analysis using the top 2000 genes with the greatest variance in expression level across all samples showed moderate clustering between pairwise time point comparisons (Fig. 3A). In comparing the ONH 7 vs 0 day samples, a total of 17,554 genes were tested for differential expression and of these only 32 were upregulated and 30 were downregulated, indicating an extremely limited transcriptional response from ONH astrocytes over this time period and confirming the LRT findings (Figs. 3B and C, Table S3). Some notable upregulated genes included (Fig. 3D): reactive astrocyte markers (*H2-T23*, *Lcn2*), a transcription factor (*STAT1*), amino acid transporters (*Slc36a2*, *Slc7a5*), apolipoprotein (*Apol9b*), an anchoring protein important for cell motility, stability and scar formation (*Akap12*), and heat shock proteins (*Hspa1b*, *Hsph1*). With very few differentially expressed genes, gene set enrichment analysis (GSEA) against the Reactome database did not find significantly enriched pathways. This was additionally confirmed against the GO and KEGG database.

Lcn2 is an acute-phase secreted protein, a supposed pan-reactive astrocyte marker, and has diverse functions including immune regulation, neuroinflammation, iron homeostasis, cell proliferation, and differentiation [39, 40]. Astrocytes are thought to be the primary source of Lcn2 in the brain and its up regulation has been noted in the retina secondary to glaucoma, implicating it in the promotion of neuroinflammation in the disease [41–47]. Lcn2 is localized to optic nerve astrocytes in optic neuritis [48], but it’s localization in the glaucomatous optic nerve is unknown. We found Lcn2 to be upregulated early (Cluster 4, Fig. 2; pairwise comparisons of 7 vs 0 days, ONH log2FC = 0.73 p.adjust value = 0.04, ONP log2FC = 2.33 p.adjust value = 1.01E-09), but surprisingly not significantly expressed at the mRNA and protein levels (Figs. S1A and B). This result highlights the heterogenous nature of the reactive astrocyte response across regions (retina vs optic nerve) and injury models.

Comparing the ONH 30 vs 7 day samples, a total of 16,826 genes were tested and of these 277 were upregulated and 164 were downregulated (Figs. 3B and C, Table S4), a larger number than between 7 vs 0 days but still a relatively small transcriptional response. Prolactin (*Prl*) was strongly downregulated in all 7 of the 30 day samples except for one, thus giving the appearance of high upregulation on the heatmap (Fig. 3D). Downregulated genes included (Fig. 3D): keratins (*Krt5*, *Krt13*, *Krt14*, *Krt17*), amino acid transporter (*Slc38a5*), heat shock protein (*Hspa1b*), collagen (*Col17a1*), ATPase transporters (*ATP10a*, *ATP10d*) and *Pecam1*. Pathway analysis showed robust upregulation of oxidative phosphorylation (primarily in the respiratory electron transport chain subunits, as observed in the LRT analysis), and RNA and protein metabolism (Fig. 3E; Table S4). Upregulation of the complex I subunit *Ndufc2* and the complex IV subunit *Cox5b* was confirmed at the protein level using immunohistochemistry (Figs. S1C-H). Many of the downregulated processes relate to astrocyte morphology (keratinization, RHO GTPase), their interaction with the surrounding extracellular matrix (collagens, integrins) and communication with each other (gap junction/connexin regulation) (Fig. 3E). These transcriptomic data are supportive of the known changes in morphology and spatial arrangement that reactive ONH astrocytes undergo in glaucoma, characterized by a general loss of processes and branching, detachment and retraction of distal processes and terminals from the circumferential surface, hypertrophy of remaining processes and in chronic cases becoming amoeboid in shape [17, 19, 49, 50]. These changes would also be expected to break the gap junctional connections between astrocytes. In addition, our data predicts a downregulation in the transport of substrates across astrocyte membranes. Two of the broader categories—vesicle-mediated transport (processes for gap junction/connexin transport and regulation) and transport of small molecules (processes for SLC-mediated, amino acids, cations/anions transport)—were unique to the ONH astrocyte response and not present in the ONP astrocyte response at any time (see below). Some, but not all of these processes recover by 30 days (see 30 vs 0 day comparison below). Subsequent GSEA on the biological process and molecular function subset of the gene ontology (GO) database confirmed these changes, many downregulated processes were associated with cell adhesion and extracellular matrix organization (Fig. S1I). Ribosomal structure was the only GO biological process upregulated (Fig. S1I), in line with the upregulation of oxidative phosphorylation. KEGG pathway analysis predictably showed an upregulation of ribosome and oxidative phosphorylation pathways and downregulation of tight junction, focal adhesion, MAPK and IL-17 signaling pathways (Fig. S1I). Leukocyte transendothelial migration was downregulated, a pathway significantly upregulated in whole ONH tissue gene expression data from the DBA/2 J mouse model of glaucoma [51].

A comparison of the ONH 30 vs 0 day sample showed many persistent transcriptional changes at 30 days, a time when IOP was declining. A total of 13,508 genes were tested and of these 599 were upregulated and 296 were downregulated, the greatest number of DEGs of all three time point comparisons (Fig. 3B, Table S5). The majority of the top 30 upregulated genes encode for ribosomal proteins (*Rps23-ps1*, *Rps29*, *Rplp2*, *Rps21*, *Rps27*, *Rpl39*, *Rps28*, *Rpl37a*, *Rps12-ps3*) and electron transport chain subunits (*Cox5b*, *Ndufc1*, *Usmg5*, *mt-Atp6*, *mt-Nd4l*)(Fig. 3D). Two noteworthy genes on the list were *mt-Rnr1* (MOTS-c) and *mt-Rnr2* (Humanin), both of which have a number of cyto- and metaboloprotective effects [52]. The vast majority of mitochondrial proteins are encoded by nuclear genes, however, both MOTS-c and Humanin are mtDNA-encoded peptides that act as “retrograde signals” relaying activity and stress signals back to the nucleus to enact changes in gene expression of nucleus-encoded mitochondrial proteins and other factors needed to maintain cellular and mitochondrial homeostasis [53, 54]. Some of the stresses known to induce this retrograde signaling include altered mitochondrial ATP production, reactive oxygen species generation and unfolded/improperly imported proteins [55], processes that were significantly upregulated in this study (see below). The top 30 downregulated genes included those for cholesterol/sterol biosynthesis (*Cyp51*, *Acat2*, *Dhcr7*, *Sqle*, *Acss2*), fatty acid biosynthesis (*Faah*), the transcription factor *Olig2*, purinergic receptor *P2ry12* and *Pdgfrb* (Fig. 3D). Processes for energy metabolism remained upregulated, particularly oxidative phosphorylation, and RNA and protein metabolism (Fig. 3F; Table S5). Other notable upregulated pathways at 30 days included ER-phagosome, endosomal/vacuolar, senescence-associated secretory phenotype (SASP) and cellular response to stimuli. Interestingly, genes encompassed in these pathways are involved in proteolysis—essential for many cellular processes of which a key one is the response to oxidative stress—and include histocompatibility complexes, anaphase-promoting complexes, ubiquitin conjugating enzymes, and proteosome subunits (Table S5). Numerous antioxidant genes were represented within the cellular response to stimuli category, including glutathione peroxidases, peroxiredoxins, superoxide dismutases and thioredoxins (Table S5). Further suggesting an important role of proteolysis, we also observed the upregulation of heat shock proteins early in injury (*Hsp90* in Fig. 2, Cluster 4; *Hspa1b* in Fig. 3D, upregulated in 7 vs 0 days), which identify misfolded or unfolded proteins and target them for proteasomal degradation. Consistent with the LRT analysis, there was persistent downregulation of cholesterol, lipid, and steroid metabolism at 30 days (Fig. 3F). However, additional downregulated pathways emerged in this pairwise comparison, including those for vesicle-mediated transport (gap junction assembly/trafficking, transport of connexons to the plasma membrane), extracellular matrix organization, cell–cell communication (adherens junction interactions) and transport of small molecules (SLC-mediated transmembrane transport). The number of pathways associated with extracellular matrix organization was reduced compared to the 30 vs 7 days comparison (Fig. 3E), suggesting that tissue remodeling programs and morphological changes associated with elevated IOP have normalized by 30 days. Supplementary GSEA on the biological process and molecular function subset of the GO database and KEGG pathway analysis confirmed these findings (Fig. S1J).

We next sought to determine upstream transcription factors associated with the reactive ONH astrocyte response at 30 days. We derived a transcription factor activity enrichment score based on the log2 fold change differential expression analysis of two groups (day 30 vs 0) and observed only limited clustering of differentially expressed transcription factors by condition (Fig. S2A). We performed motif analysis with HOMER to search for existing motifs that were enriched in regions flanking the transcription start site (TSS) of an input list of genes; here we focused on significantly upregulated genes (adjusted *p*.value < 0.05, log2FC > 0, motifs searched within a region of ± 2 kb around the TSS). Five motifs and their corresponding transcription factors were found to be enriched (ETS, YY1, ELF1, Elk1, Elk4, Fig. S2B).

We previously showed that cocaine and amphetamine-regulated transcript (*Carpt*) is an ONH astrocyte specific marker [33]. Here we found that it was not differentially expressed at any time following IOP elevation (7 vs 0 days, log2FC = 0.02, p.adjust 0.95; 30 vs 7 days, log2FC = 0.03, p.adjust 0.92; 30 vs 0 days, log2FC = 0.12, p.adjust 0.54; Tables S3, 4 and 5). It was the top differentially expressed gene in ONH vs ONP comparison at 30 days (Fig. S2C), and its in-situ and immunohistochemical localization remained within ONH astrocytes at 7 and 30 days (Fig. S2D and E). The results indicate that *Cartpt* remains a specific marker of ONH astrocytes following IOP elevations.

### Optic nerve proper astrocytes show a greater transcriptional response to elevated intraocular pressure than optic nerve head astrocytes and they also primarily upregulate oxidative phosphorylation

**Fig. 4:**
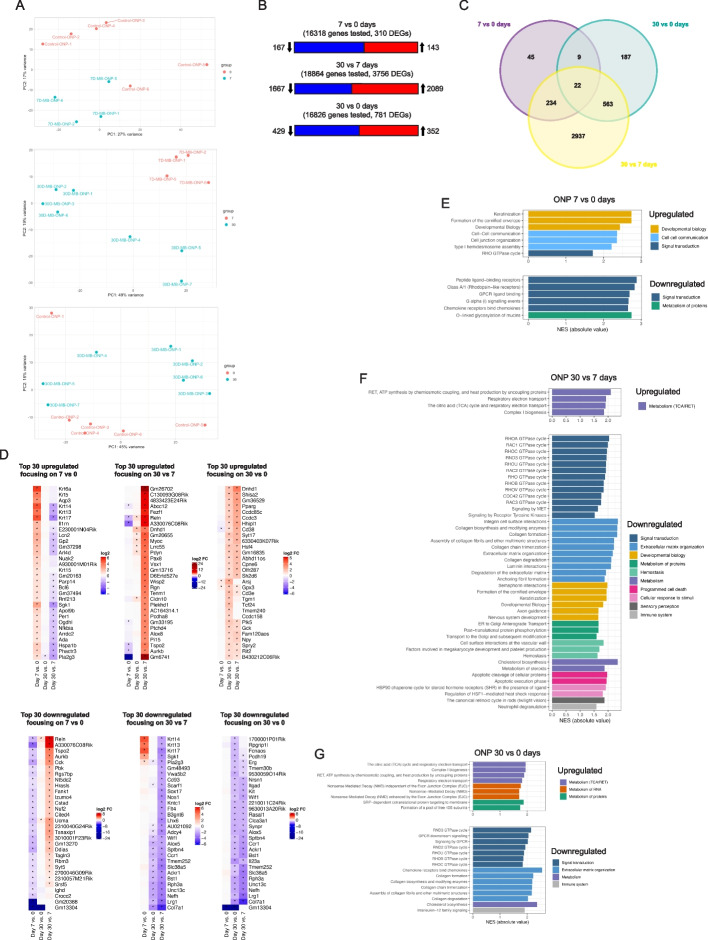
Transcriptional response of optic nerve proper astrocytes to elevated IOP. **A** Principal component analysis based on expression levels of the top 2000 most variable genes across 7 vs 0, 30 vs 7 and 30 vs 0 day ONP samples. **B** Number of DEGs for each independent time point pairwise comparison. Significance was adjusted *p*-value < 0.05; no log2 fold change cutoff was applied. **C** Venn diagram showing the number of unique and overlapping DEGs across the 3 independent time point comparisons (adjusted *p*-value < 0.05). **D** Top 30 up and downregulated genes for each of the 3 independent time point comparisons, based on rank from largest to lowest log2 fold change value (absolute value, log2 FC > 0 upregulated, log2 FC < 0 downregulated). **E** Up and downregulated pathways from the 7 vs 0 day sample. Based on over-representation analysis of the up and down regulated genes using the Reactome database. Listed are all pathways with adjusted *p*-value < 0.05. Each pathway is color matched for the broader category it belongs to. NES refers to the normalized enrichment score that accounts for differences in gene set size and in correlations between gene sets and the expression dataset. **F** Up and downregulated pathways from the 30 vs 7 day sample. Based on over-representation analysis of the up and down regulated genes using the Reactome database. Listed are all pathways with adjusted *p*-value < 0.05. Each pathway is color matched for the broader category it belongs to. **G** Up and downregulated pathways from the 30 vs 0 day sample. Based on over-representation analysis of the up and down regulated genes using the Reactome database. Listed are all pathways with adjusted *p*-value < 0.05. Each pathway is color matched for the broader category it belongs to

Optic nerve pathology in glaucoma preferentially affects the nerve head tissue region and we hypothesized that astrocytes in the myelinated ONP region would behave differently to those in the ONH region. Principal component analysis using the top 2000 genes with the greatest variance in expression level across all samples showed clear separation for the 30 vs 7 day samples (Fig. 4A). Based on the number of DEGs between the three independent pairwise time point comparisons, ONP astrocytes showed a greater transcriptional response than ONH astrocytes (Fig. 4B). Although both have approximately the same number of DEGS at 30 days (895 vs 781), ONP astrocytes experienced a greater early change (7 vs 0 days) and during the remainder of the injury period (30 vs 7 days). Comparing the 7 vs 0 day samples there were 310 DEGs (contrasting with only 62 in ONH astrocytes; Figs. 3B and C, Table S6), 143 were upregulated and 167 downregulated. Of the top 30 upregulated genes many were keratins (*Krt5, 6a, 13, 14, 17*), and several were in common with the top 30 upregulated genes in ONH astrocytes (*Lcn2*, *Apol9b*, *Hspa1b*) (Fig. 4D). Unlike the ONH astrocyte response at this time, pathway analysis showed differential regulation of several processes (Fig. 4E; Table S6). Keratins are intermediate filaments that function as part of the cytoskeleton to mechanically stabilize cells against physical stress. This occurs through connections to desmosomes and hemidesmosomes. Keratinization and cornification increase keratin production, which is in line with the view that astrocytes experience mechanical stress following elevated IOP; however, it was surprising that an early increase was observed in ONP and not ONH astrocytes. Keratinization and cornification were downregulated in both ONH and ONP (see below) astrocytes between 7 and 30 days, indicating that strengthening was only required early when IOP was rising. Genes annotated for cell–cell communication mediate the formation and maintenance of adherens junctions, tight junctions, as well as aspects of cellular interactions with the extracellular matrix and hemidesmosome assembly.

The greatest number of gene expression changes was observed in the 30 vs 7 day comparison where there were 3756 DEGs (2089 upregulated and 1667 downregulated; Figs. 4B and C, Table S7). Optic nerve proper astrocytes demonstrated 22 genes differentially expressed in all three timepoint comparisons suggesting a common set of injury response genes (Fig. 4C; Table S8). The top 30 up and downregulated genes are shown in Fig. 4D. Like ONH astrocytes, oxidative phosphorylation was a key upregulated process, notably the only one (Fig. 4F, Table S7). There were a large number of downregulated processes, and of these, extracellular matrix organization and developmental biology were downregulated at this time point comparison in both ONH and ONP astrocytes (Fig. 4F).

A comparison of the 30 vs 0 day sample discovered 781 DEGs (352 upregulated and 429 downregulated; Fig. 4B, Table S9). Among the top 30 upregulated genes were heat shock transcription factor 4 (*Hsf4*), glutathione peroxidase 3 (*Gpx3*), neuropeptide Y (*Npy*), and glucokinase (*Gck*). Top downregulated genes included: collagen (*Col7a1*), WNT inhibitory factor 1 (*Wif1*), RAS protein activator like 1 (*Rasal1*), arachidonate 5-lipoxygenase (*Alox5*), C–C motif chemokine receptor (*Ccr1*), interleukin 23 subunit alpha (*Il23a*), and solute carrier family 38 member 5 (*Slc38a5*). The large reduction in DEGs compared to the 30 vs 7 day comparison indicates many processes are likely to have stabilized by 30 days, and this is also reflected in the reduced number of differentially regulated pathways (Fig. 4G). In all, there were several commonalities and differences in the ONH vs ONP astrocyte response to elevated IOP at 30 days, indicating unique functional specializations in the response of different astrocyte populations in the optic nerve. In common, both ONH and ONP astrocytes upregulate oxidative phosphorylation and RNA and protein metabolism (30 vs 0 days), however, ONH astrocytes additionally upregulated antioxidative capacity and proteolysis. Only ONH astrocytes downregulated processes for vesicle-mediated transport (gap junction/connexon trafficking and regulation) and transport of small molecules (SLC-mediated, amino acids, cations/anions). Although both ONH and ONP astrocytes downregulated cholesterol biosynthesis, ONH astrocytes also downregulated other lipid pathways, including steroid, phospholipid and glycerophospholipid metabolism.

### Elevated intraocular pressure increases ATP production, mitochondrial biogenesis and shifts the cellular source of ATP

**Fig. 5:**
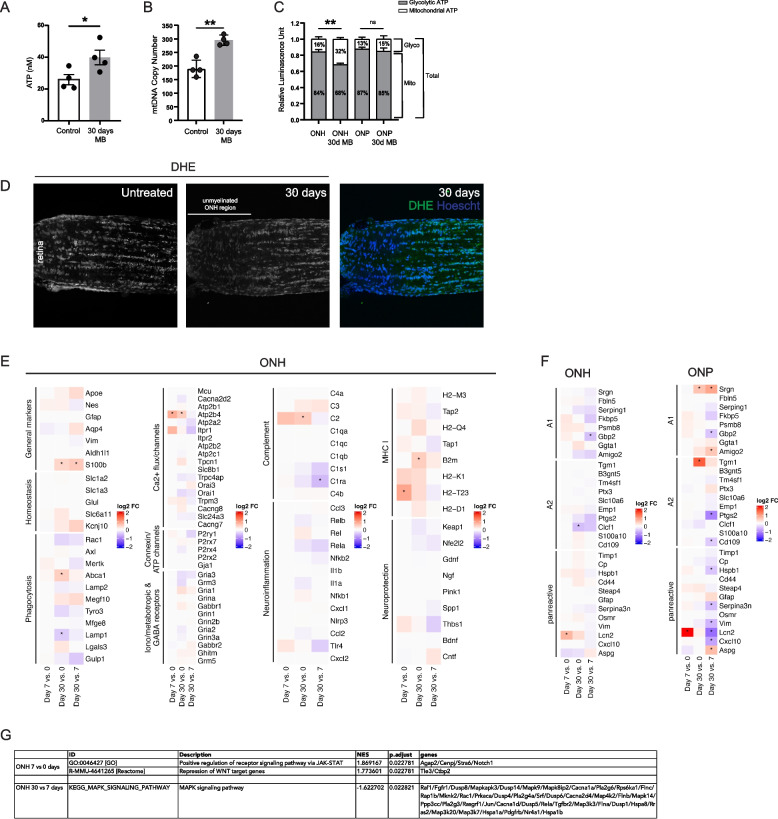
Expression of various astrocyte genes and signaling pathways. **A** Functional ATP assay measuring ATP levels in optic nerve head tissues from control and 30 days microbead injected animals. MB = microbead injected. *N* = 4 for each group. Mean ± SEM. * *p* < 0.05. **B** Mitochondrial biogenesis in optic nerve head tissues from control and 30 days microbead injected animals. MB = microbead injected. *N* = 4 for each group. Mean ± SEM. ** *p* < 0.01. **C** Changes in the proportion of cellular ATP derived from glycolysis versus oxidative phosphorylation in response to elevated IOP within optic nerve head tissues. Oligomycin was used to inhibit ATP synthesis by oxidative phosphorylation. *N* = 4 for each group. Mean ± SEM. *** *p* < 0.01. **D** Reactive oxygen species was measured using DHE (dihydroethidium) labeling of live unfixed longitudinal sections of the optic nerve. **E** Log2 fold change values for various astrocyte genes, as determined in the differential expression analysis of the 3 independent time point pairwise comparisons. Astericks indicate significant differences between the 2 groups (adjusted *p*-value < 0.05). **F** Log2 fold change values for reactive astrocytes genes, as determined in the differential expression analysis of the 3 independent time point pairwise comparisons. Astericks indicate significant differences between the 2 groups (adjusted *p*-value < 0.05). **G** Gene set enrichment analysis on the 3 time point pairwise comparisons against a user defined subset of the GO, KEGG and Reactome pathway, focusing on key reactivity and injury signaling pathways: nuclear factor kappa B (NFkB), calcineurin-nuclear factor activating of T-cells (CaN-NFAT), mitogen-activated protein kinase (MAPK), janus kinases/signal transducer and activator of transcription (JAK/STAT), Wnt/b-catenin, and sonic hedgehog (adjusted *p*-value < 0.05). NES = normalized enrichment score

Along with the transcriptional upregulation of oxidative phosphorylation, ONH astrocytes showed an increase in ATP production (Fig. 5A) and mitochondrial biogenesis (Fig. 5B). Using an assay to measure glycolysis and oxidative phosphorylation we observed a metabolic shift in cellular ATP source in response to elevated IOP; ONH astrocytes significantly doubled the proportion of ATP derived from oxidative phosphorylation (from 16 to 32%) and decreased the amount from glycolysis (from 84 to 68%; Fig. 5C), consistent with the transcriptional observations. Interestingly, there was no such shift in ONP astrocytes following elevated IOP (Fig. 5C). Although we use whole tissues for these assays, as the optic nerve head and proper regions are densely populated with astrocytes it is most likely the results reflect changes in astrocyte metabolism. Oxidative phosphorylation also increases the production of reactive oxygen species and ONH astrocytes upregulated defense genes including glutathione peroxidases, peroxiredoxins, superoxide dismutases and thioredoxins (Fig. 3F and Table S5). We used a DHE (dihydroethidium) assay kit to measure reactive oxygen species directly in live unfixed cells at 30 days and indeed found the levels to be significantly lower in all cells of the ONH region compared to the ONP, consistent with an upregulation in the defense gene activity (Fig. 5D).

### Genes and pathways that differentiate the optic nerve head astrocytes from those in the optic nerve proper are largely retained following elevated intraocular pressure

We wanted to determine whether the transcriptional profile that differentiated ONH astrocytes vs those in the ONP was retained following elevations in IOP. We compared 30 day ONH samples with matching ONP samples and found that genes and pathways distinguishing ONH vs ONP astrocytes at 0 day [33] continued to be high differentially expressedø in the 30 day comparison. Numerous top 50 genes in the ONH vs ONP comparison at 0 days were also found at 30 days, including: *Cartpt*, *Mgarp, Dct*, *Tes*, *Clec18a*, *Cntnap2*, *Tmem37*, *Bok*, *Ankrd33b*, *Col8a1* (compare Fig. S2C with Fig. 3B in [33]). Similarly, oxidative phosphorylation, metabolism of protein, RNA and extracellular matrix organization differentiated ONH vs ONP astrocytes in both 0 and 30 days (Table S10). This indicates that regional astrocyte identity is largely maintained in elevated IOP.

### Optic nerve head astrocytes show a limited neuroinflammatory transcriptional response following elevated intraocular pressure

Having examined genes and pathways differentially expressed using a global, unbiased approach, we next looked at genes of interest related to general astrocyte biology, including canonical astrocyte markers and genes involved in astrocyte homeostasis. We looked at the cytoskeletal markers *Gfap*, *Vim* and *Nes*, the cytosolic markers *Aldh1l1*, *Aldoc*, *S100β*, the lipoprotein *Apoe*, and the water channel *Aqp4* (Fig. 5E). All were not significantly differentially expressed at any time point comparison except for *S100 β * which showed upregulation at 30 days (30 vs 7; log2FC 0.53, p.adjust 0.03; 30 vs 0; log2FC 0.42, p.adjust 0.03). Similarly, very few genes associated with the general function of astrocytes (homeostasis, phagocytosis, Ca^2+^ flux, connexins/ATP channels, receptors) were differentially expressed (Fig. 5E). *Abca1* was upregulated at 30 days (log2FC 0.48, p.adjust 0.02) and is associated with numerous functions such as (1) the efflux of cholesterol and phospholipids to lipid-free apolipoproteins, (2) phagocytosis, although its pathway molecules *Megf10* and *Gulp1* were not upregulated, (3) immune cell regulation and (4) anti-inflammation. *Lamp1* is a lysosomal membrane protein and was downregulated at 30 days (log2FC -0.44, p.adjust 0.03). *Atp2b4* is a plasma membrane Ca^2+^-transporting ATPase and was upregulated at both 7 (log2FC 0.82, p.adjust 0.04) and 30 days (log2FC 0.51, p.adjust 0.02). The ONH is densely populated with astrocytes and is a site of neuroinflammatory changes in glaucoma. Of the genes related to complement, neuroinflammation and MHC I very few were upregulated (Fig. 5E). *C2* was upregulated at day 30 (log2FC 0.53, p.adjust 0.0004) and *C1ra* downregulated (log2FC -0.58, p.adjust 0.04). *C3*, a general marker of astrocyte reactivity [56] was not differentially expressed, but *H2-T23* was at 7 days (log2FC 0.89, p.adjust 0.01). Select genes for neuroprotection were also not differentially expressed (Fig. 5E).

Particular gene signatures have been identified as being associated with whether the reactive astrocyte response is purportedly damaging (A1 phenotype), purportedly supportive (A2 phenotype) or pan-reactive [56]. Although there are caveats to using this classification system (see Discussion) we have nonetheless provided this analysis as a framework to allow comparison with the many other studies that have already used the nomenclature. Our results showed few genes significantly differentially expressed, with no bias towards any one phenotype (Fig. 5F). Of note, *Lcn2* was an early (7 vs 0 days) upregulated gene in both ONH (log2FC 0.74, p.adjust 0.04) and ONP (log2FC 2.34, p.adjust 1.01 × 10^–9^) astrocytes.

Finally, we used GSEA to ask whether key astrocyte signaling pathways involved in reactivity and the injury response were enriched following elevated IOP. We restricted this follow-up GSEA to six pathways: nuclear factor kappa B (NFkB), calcineurin-nuclear factor activating of T-cells (CaN-NFAT), Mitogen-Activated protein kinase (MAPK), Janus kinases/signal transducer and activator of transcription (JAK/STAT), Wnt/b-catenin, and sonic hedgehog, sourced from the GO, KEGG, and Reactome databases. Examining the results from pairwise time point comparison for ONH and ONP astrocytes we found differential expression in a limited number of pathways and only for ONH astrocytes (Fig. 5G). Repression of WNT target genes and positive regulation of receptor signaling pathway via JAK-STAT were significantly upregulated at 7 days. MAPK signaling was significantly downregulated at 30 days.

**Fig. 6:**
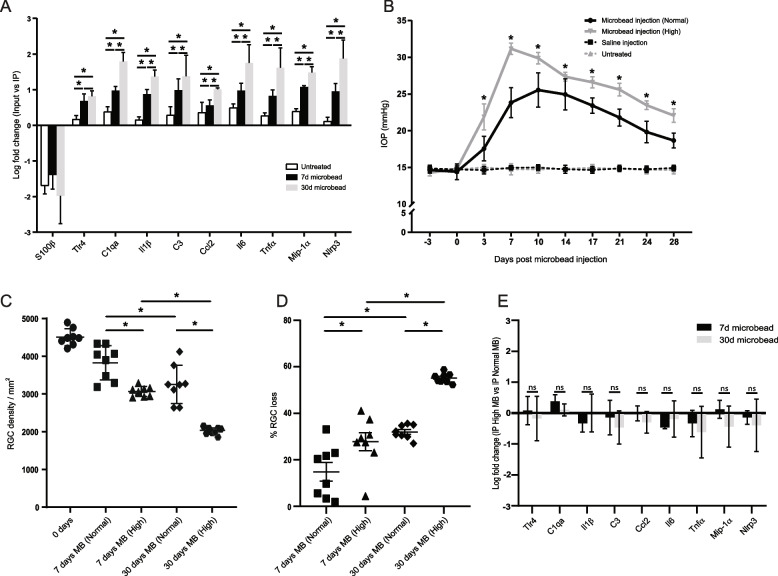
Optic nerve head astrocytes show a limited neuroinflammatory transcriptional response. **A** RT**-**qPCR of the ONH input vs immunoprecipitate (IP) from untreated, 7 and 30 day microbead injected mice for select glaucoma-related neuroinflammatory genes. *N* = 4 per group. Mean ± SEM. * *p* < 0.05. **B** IOP measures over the 30 day experimental period. Same as in Fig. 1B, with the addition of IOP measurements from a cohort of mice that were injected with a larger concentration and volume of microbeads (High). *N* = 6 for each group. Mean ± SEM. * *p* < 0.01 (for the comparison between High vs Normal microbead injection). **C** Loss of ganglion cell density in the treated eye. Same as in Fig. 1C, with the addition of counts from a cohort of mice that were injected with a larger volume of microbeads (High). *N* = 8 biological replicates. * *p* < 0.05. Paired students t-test. **D** Loss of ganglion cells in the treated eye as a percentage of the untreated contralateral eye. Same as in Fig. 1C, with the addition of percentage loss from a cohort of mice that were injected with a larger volume of microbeads (High). *N* = 8 biological replicates. * *p* < 0.05. Paired students t-test. **E** RT-qPCR of the ONH immunoprecipitate (IP) from mice that were injected with the larger amount of microbead (High) vs those that were injected the normal amount (Normal) and tested for select glaucoma-related neuroinflammatory genes. *N* = 4 per group. Mean ± SEM. ns = not significant. Paired students t-test

As neuroinflammation related transcriptional changes have been reported in the ONH region [7–10, 57, 58] and we did not observe differential expression of many neuroinflammatory genes in ONH astrocytes, we hypothesized that other cells within the ONH tissue account for the observation. We used RT-qPCR and compared the expression of select genes between the ONH Input vs IP fraction (Fig. 6A). Indeed, we found that at all time points (0, 7 and 30 days) the Input fraction was significantly more enriched for neuroinflammatory genes compared to the IP, suggesting that non-astrocytes such as microglia upregulate these genes. Moreover, for each gene the enrichment in the Input fraction increased significantly over time (Fig. 6A).

The reactive astrocyte response is context-dependent and can vary with the severity of injury. We wanted to determine if a greater elevation in IOP could induce a differential neuroinflammatory transcriptional response in ONH astrocytes. We injected a greater volume of microbeads (4.01 × 10^7^ beads/ml), leading to a higher peak IOP, a more prolonged elevation in IOP and greater ganglion cell loss (Figs. 6B-D). We compared the high microbead IP vs normal microbead IP at each timepoint and found that for most genes a high microbead injection decreased the expression of neuroinflammatory genes in ONH astrocytes (Fig. 6E). There was no significant difference between 7 and 30 days for all genes.

## Discussion

The reactive response of optic nerve astrocytes in glaucoma is not well understood. Here, we have used astrocyte-ribotag, followed by bulk RNA-seq to determine the global transcriptional response of ONH and ONP astrocytes to a microbead induced elevation in IOP. Ribotag immunoprecipitation enabled efficient isolation of astrocyte-specific ribosomal mRNA from small complex tissues in vivo. Ribosomal mRNA more closely reflects the proteins being functionally used by the cell, or the active translational state of the cell, rather than the whole transcriptome irrespective of functional relevance [31, 32]. As well, the sequencing is weighted toward more frequently translated transcripts. This approach also isolates mRNA being translated in distal processes; a population of mRNAs often lost using other methods. To our knowledge, this is the first study assessing the astrocyte-specific response to elevations in IOP as prior transcriptional studies used whole optic nerve tissues, which does not allow easy identification of the cells responsible for the differential expression. We demonstrate that astrocytes of the optic nerve exhibit a region-specific and temporally distinct transcriptional response to elevated IOP, further highlighting that the ONH represents a complex microenvironment with different glial support requirements from the neighboring myelinated region. These results allow us to associate transcriptional programs with the morphological changes we have previously described [17–19].

The ONH environment is unique in many ways; not only do ganglion cell axons have high signaling activity and thus metabolism, in this region they are unmyelinated; the tissue surrounding the region undergoes extensive biomechanical strain from diurnal variations in IOP and eye movements; the vascular network is denser, more complex and has a leakier blood brain barrier than in the myelinated ONP, and there is a higher density of mitochondria [11, 14, 15, 59, 60]. These features are reflective of a region that has a high energy demand, but is subsequently particularly vulnerable to energy insufficiency. The upregulation of energy metabolism, particularly oxidative phosphorylation emerged as a key response of ONH astrocytes to elevated IOP. Astrocytes were traditionally considered to predominantly use the glycolytic pathway for production of ATP [61, 62], which, although yielding minor amounts of ATP, produces lactate which can then be shuttled to neurons as an energy substrate (astrocyte-neuron lactate shuttle) [63–66]. Accordingly, oxidative phosphorylation was regarded as of minor importance. This is no longer thought to be the case as recent studies show that extracellular K^+^ buffering – the most archetypal homeostatic task of astrocytes – is not just passive (e.g., diffusion through Kir 4.1 channels and gap junctions), but also mediated by the energy demanding Na^+^-K^+^ ATPase pumps [67–69]. This pump is also the major pathway for astrocytes to remove excess Na^+^ that accumulates in its role of clearing neurotransmitters. Such energy demands cannot be met by glycolysis which produces 2 molecules of net ATP per molecule of glucose, compared to the 36 produced via oxidative phosphorylation. New estimates of energy consumption that consider the role of the Na^+^-K.^+^ pump reveal that astrocytes consume up to 37% of the total CNS energy budget (more than 200% higher than the previously estimated 7%), and more than 75% of astrocytic ATP is derived from mitochondrial oxidative phosphorylation [67, 70]. This does not negate the importance of glycolysis, as it remains a key pathway for astrocytes to provide axons with energy substrates. For example, Cooper et al., 2020 [71] recently discovered that astrocyte networks driven by Cx43 redistribute glucose from the unstressed eye to the stressed eye in response to elevated IOP. In our study, the reason for a robust upregulation of oxidative phosphorylation may be that astrocytes require greater energy when reactive in an effort to maintain the normal functioning of ‘homeostatic’ processes that are essential in times of stress, including glycogen storage and mobilization, supply of energy substrates and precursors for biosynthesis, phagocytosis and transmitophagy, membrane trafficking, cytoskeletal remodeling and proteolysis that involves the ATP-dependent chaperones and proteases (such as heat shock proteins which were upregulated in our study). In other words, astrocytes look out for themselves before they help others, similar to reactive gray matter astrocytes which have been described as ‘selfish’ [72]. It is also interesting to consider if ONH astrocytes can transfer healthy mitochondria to axons. Just as neurons have been shown to utilize astrocytes for elimination of degraded mitochondria (transmitophagy [73];), evidence in a mouse model of stroke has shown that astrocytes donate healthy mitochondria to neurons after injury as a way to improve their survival [74]. It remains unknown whether a similar behavior occurs in the optic nerve; although our data shows an increased expression of mitochondrial components by astrocytes in this region that would be consistent with such a phenomenon, we are unable to conclusively demonstrate this possibility. Such a process would make sense in the ONH region because there is a high density of mitochondria that requires constant turnover, and defects in axonal transport emerge early in the disease preceding somatic degeneration [75–78]. In DBA/2 J mice, anterograde transport is affected earlier than retrograde, with transport dysfunction proceeding distally from the superior colliculus to the optic nerve then retina, while key RGC structures in the superior colliculus remain [75]. Defects in transport would be expected to result in an accumulation of damaged mitochondria. Compounding this, astrocytes may have a more limited phagocytic capacity than microglia which are professional phagocytes, and the need to eliminate axonal debris and damaged mitochondria as glaucoma progresses may exceed this capacity and result in a ‘backlog’ of damaged mitochondria. In this scenario, ONH astrocytes would provide healthy mitochondria and be involved in transmitophagy. Our results showed an increase in mitochondrial biogenesis within the ONH region. Very interestingly, resident microglia also strongly upregulate oxidative phosphorylation and not other pathways in the DBA2/J mouse model of glaucoma. Neuroinflammation was downregulated [79]

An inevitable by-product of oxidative phosphorylation is the production of reactive oxygen species which subsequently damages cellular proteins through oxidation [80]. As a response to counteract these processes ONH astrocytes upregulated antioxidative capacity and proteolysis. Astrocytes have a primary role in providing antioxidant support to surrounding neurons through several systems that include the glutathione system, thioredoxin/peroxiredoxin system, superoxidase dismutases, and catalase [81]. They are the primary producers of and can store glutathione [82]. Our data showed an upregulation of the cellular response to chemical stress pathway, encompassing genes for glutathione peroxidase (*Gpx1*, *Gpx3*), peroxiredoxin (*Prdx1*, *Prdx2*, *Prdx5*), superoxide dismutase 1 (*Sod1*), and thioredoxin (*Txn1*). These genes play an important role in the catabolism of peroxide (H_2_O_2_) and superoxide anions (O_2_^−^). The central redox-sensor *Nrf2/Nfe2l2* was not differentially expressed at any time, indicating the increase in ROS could be managed by antioxidative systems and was not above “normal” (physiological) levels [83]. The ability of cells to restore proteins after oxidative damage is limited and, in most cases, damaged proteins are removed via proteolysis, mediated by either the ubiquitin-proteasomal or the lysosomal system. The ubiquitin-proteasomal system is responsible for the proteolytic degradation of 80–90% of all cellular proteins, including many regulated, short-lived, or misfolded/damaged ones [80]. We found an upregulation of genes encompassing histocompatibility complexes, anaphase-promoting complexes, ubiquitin conjugating enzymes, and proteosome subunits. It was surprising to see the upregulation of proteolysis at the same time as protein metabolism, as cells typically downregulate global protein synthesis with increased levels of reactive oxygen species [80]. The increased antioxidative capacity and proteolysis were unique to the ONH astrocyte response and not seen in the ONP astrocyte response at any time, even though they also upregulated oxidative phosphorylation.

It was surprising that ONH astrocytes demonstrated a largely late transcriptional response, with very few DEGs before 7 days. Astrocytes are generally thought to be sensitive to stresses and ONH astrocytes are localized to a region prone to continuous biomechanical stress. One explanation could be that because of their location they are naturally more resistant to stresses and strains and require a greater severity of injury before significant transcriptional changes are observed, one that is not reached until 7 days in our model. This seems to be consistent with our previous study in which sub-threshold increases in IOP induced a reversible reactive morphological change in the absence of transcriptional changes (measured using microarray) [19]. Moreover, healthy uninjured ONH astrocytes are fortified and strengthened by expressing higher levels of cytoskeletal and extracellular matrix components than ONP astrocytes [33, 49]. It is also important to note that although ONH astrocytes are morphologically similar [14], not all become reactive simultaneously [84], and bulk RNA-seq likely masks reactive changes of individual astrocytes. In this light, ONP astrocytes which are de-enriched in cytoskeletal components [33] and are not as necessarily resilient demonstrate a more significant early transcriptional change, interestingly, an upregulation in processes that suggest they are stiffening their cytoskeleton (keratinization). Another possibility is that because the myelinated ONP contains more microglia than the ONH (at least early in injury) they are more likely to receive an early microglial signal, if it is that microglia respond first in white matter region [85]. At 30 days, ONH astrocytes showed persistent transcriptional changes. This could arise from IOP-dependent and/or independent mechanisms such as: (1) the IOP still being elevated, (2) it could reflect a new “normal” state for these astrocytes irrespective of IOP, (3) RGC axon degeneration will strongly influence the transcriptional profile irrespective of whether the IOP continues to decline or remain elevated, and (4) astrocyte interactions with infiltrating macrophages or microglia. It is likely that a combination of these have a role in the observed transcriptional profile and our study cannot differentiate between these.

Neuroinflammation within the ONH region is recognized as a key component of glaucomatous neurodegeneration, although the exact contribution from astrocytes is not well understood [7, 9, 10]. This is in part because many transcriptomic studies use whole ONH tissues and some even have possibly included unknown amounts of myelinated ONP, therefore precluding cellular localization of the changes in expression – the ONH also contains microglia, axons, endothelial cells, and very few oligodendrocytes and NG2 glial cells. This becomes particularly challenging when experimental glaucoma is induced as early monocyte infiltration can significantly contribute to a pro-inflammatory environment in the ONH [23, 24]. Tribble et al. [79] discovered that in the DBA2/J mice, resident microglia show few changes in neuroinflammatory molecules but are metabolically active, and that the neuroinflammatory ONH environment can be attributed to infiltrating macrophages. In general, transcriptional studies using whole ON tissues have demonstrated an upregulation of cellular pathways for the complement cascade, IL6, cell proliferation, Toll-like receptors, *Tnfα*, *Wnt*, *Tgfβ* and *Nfkβ* [23, 24, 51, 57, 58, 86–90]. Astrocyte-specific studies employing immunohistochemistry, cell cultures or transgenic manipulations have confirmed some of the findings, albeit at the level of individual molecules. To get a more accurate picture of the global transcriptional changes specific to astrocytes we used the ribotag approach.

Surprisingly, at no time did we observe a robust neuroinflammatory response from optic nerve astrocytes. Very few glaucoma-related neuroinflammatory and reactive astrocyte genes and pathways were differentially expressed (*Hsp70*, *Hsp90*, *Hspb1*, *C2*, *Tlr4*, *S100b*, *Lcn2*, *H2-T23*), and understanding their significance to an overall neuroinflammatory astrocyte requires more focused functional testing. Of note are the heat shock proteins. Heat shock proteins are upregulated in the retina and ONH of humans with the disease as well as in monkey and rodent models (HSP27, HSP60; [91–97]). Although many studies largely implicate RGCs as a target for heat shock protein-driven autoimmunity, our results potentially suggests that astrocytes could also be impacted, particularly via HSP70. Because the reactive response can depend on the severity of injury [21], we also performed a more severe elevation in IOP and assessed the expression of several established glaucoma-related neuroinflammatory genes. For virtually all the genes assessed, the higher elevation in IOP model either minimally changed or even decreased the expression of the genes compared to the normal elevation in IOP model, further supporting their limited neuroinflammatory response in this context. Interestingly, a more focused analysis of key signaling pathways involved in the astrocyte injury response showed upregulation of receptor signaling via JAK-STAT. STAT3, a member of the JAK-STAT signaling family is known to be a critical regulator of astrocyte reactivity and is mostly considered to mediate the supportive functions of reactive astrocytes in numerous disorders [98, 99]. In glaucoma, we previously showed that attenuation of STAT3-mediated astrocyte reactivity leads to a worse outcome for retinal ganglion cell survival and visual function [17]. Our results also demonstrated very few differentially expressed A1, A2 pan-reactive astrocyte marker genes and overall, there was no bias towards any one phenotype. This is consistent with recent studies [84], and with the growing consensus that this “binary polarization” of reactive astrocytes in either neurotoxic or neuroprotective states is too simplistic and falls short of fully recognizing their phenotypic diversity across disorders [56]. Moreover, the classification was defined based on two very disparate acute injury models and on the response from gray matter cortical astrocytes, how this translates to white matter astrocytes is unknown. There are additional concerns with the classification, and these have been well described in the recent astrocyte consensus statement [56]. Further confirming the limited neuroinflammatory role of astrocytes, we observed that the Input fraction – which contains all other cellular components of the ONH—was far more enriched for neuroinflammatory genes than the IP fraction, inferring that cells in the Input, likely the microglia, are contributing most to the neuroinflammatory ONH environment.

We previously showed that reactive ONH astrocytes have a beneficial role in an acute and microbead model of elevated IOP as attenuating reactivity enhanced ganglion cell degeneration and functional loss [17]. Our data here supports this notion and further indicates that regulation of their own metabolism, particularly oxidative phosphorylation, redox homeostasis and the maintenance of a healthy proteome are key factors in their support to axons. Dai et al. [49] proposed that it may not be the IOP-related mechanical stress upon axons, but rather the retraction of astrocyte processes from axons they normally ensheath, that ultimately damage axons in the glaucomatous ONH region, highlighting the importance of astrocytic-axon metabolic support. These results suggest astrocytic metabolism rather than neuroinflammation as a target for improved neuroprotection. Our data also provides a new unique resource to help drive further hypothesis generation and testing in glaucoma.

### Supplementary Information

**Additional file 1: Supplementary Figure 1.** (A-B) In-situ hybridization (A, A’) and immunohistochemical staining (B) of the mouse optic nerve for LCN2. We observed very sparse labeling of LCN2 at 7 days after microbead injections (arrowheads). (C-H) Longitudinal sections of the mouse ONH region immunostained for NDUFC2 and COX5B in untreated and microbead injected mice at 30 days. (I, J) Gene set enrichment analysis using the gene ontology (GO) and KEGG database.

**Additional file 2: Supplementary Figure 2.** (A) Heatmap showing the per-sample activity score for each transcription factor identified as significantly different between ONH 30 vs 0 days.  While there are significant differences based on the log2 fold change of genes as input, the heatmap showed limited clustering by condition. (B) Table summarizing the known motifs found to be enriched in the transcription start site +/- 2kb region of our input gene list, with a p-value < 0.001.  For each known motif, the corresponding transcription factor is specified. (B) Table summarizing the known motifs found to be enriched in the transcription start site +/- 2kb region of our input gene list, with a p-value < 0.001.  For each known motif, the corresponding transcription factor is specified. (D) In-situ hybridization showing the localization of *Cartpt* mRNA in the optic nerve of a microbead injected mouse at 30 days. (E) Longitudinal sections of the mouse ONH region showing immunostaining for CARTPT and SOX9 in microbead injected mice at 30 days.

**Additional file 3: Supplementary Table 1.** The quantity and quality of RNA and cDNA measured using the Agilent 2100 Bioanalyzer.

**Additional file 4: Supplementary Table 2.** Differential expression analysis using a likelihood ratio test (LRT) focusing on genes in the ONH that differ over time (0, 7 and 30 days) in microbead injected mice.

**Additional file 5: Supplementary Table 3.** Differentially expressed genes in pairwise comparisons of ONH 7 vs 0 days.

**Additional file 6: Supplementary Table 4.** Differentially expressed genes and significant pathways identified in ONH 30 vs 7 days.

**Additional file 7: Supplementary Table 5.** Differerentially expressed genes and significant pathways identified in ONH 30 vs 0 days.

**Additional file 8: Supplementary Table 6. **Differentially expressed genes and significant pathways identified in ONP 7 vs 0 days.

**Additional file 9:  Supplementary Table 7.** Differentially expressed genes and pathways identified in ONP 30 vs 7 days.

**Additional file 10: Supplementary Table 8.** Common differentially expressed genes in all three timpoint comparisons in the ONP.

**Additional file 11: Supplementary Table 9.** Differentially expressed genes and pathways identified in ONP 30 vs 0 days.

**Additional file 12: Supplementary Table 10.** Pathways differentiating ONH astrocytes from those in the ONP at 30 days.

## Data Availability

The dataset supporting the conclusions of this article have been deposited at GEO (GSE237073 microbead injected animals. GSE211515 normal untreated animals) and publicly available at the date of publication.
