# Supplementary figures and images for: Astrocytes of the optic nerve exhibit a region-specific and temporally distinct response to elevated intraocular pressure
Source: Mol Neurodegener. 2023 Sep 27;18:68. doi: 10.1186/s13024-023-00658-9 (PMC10523752; doi:10.1186/s13024-023-00658-9)

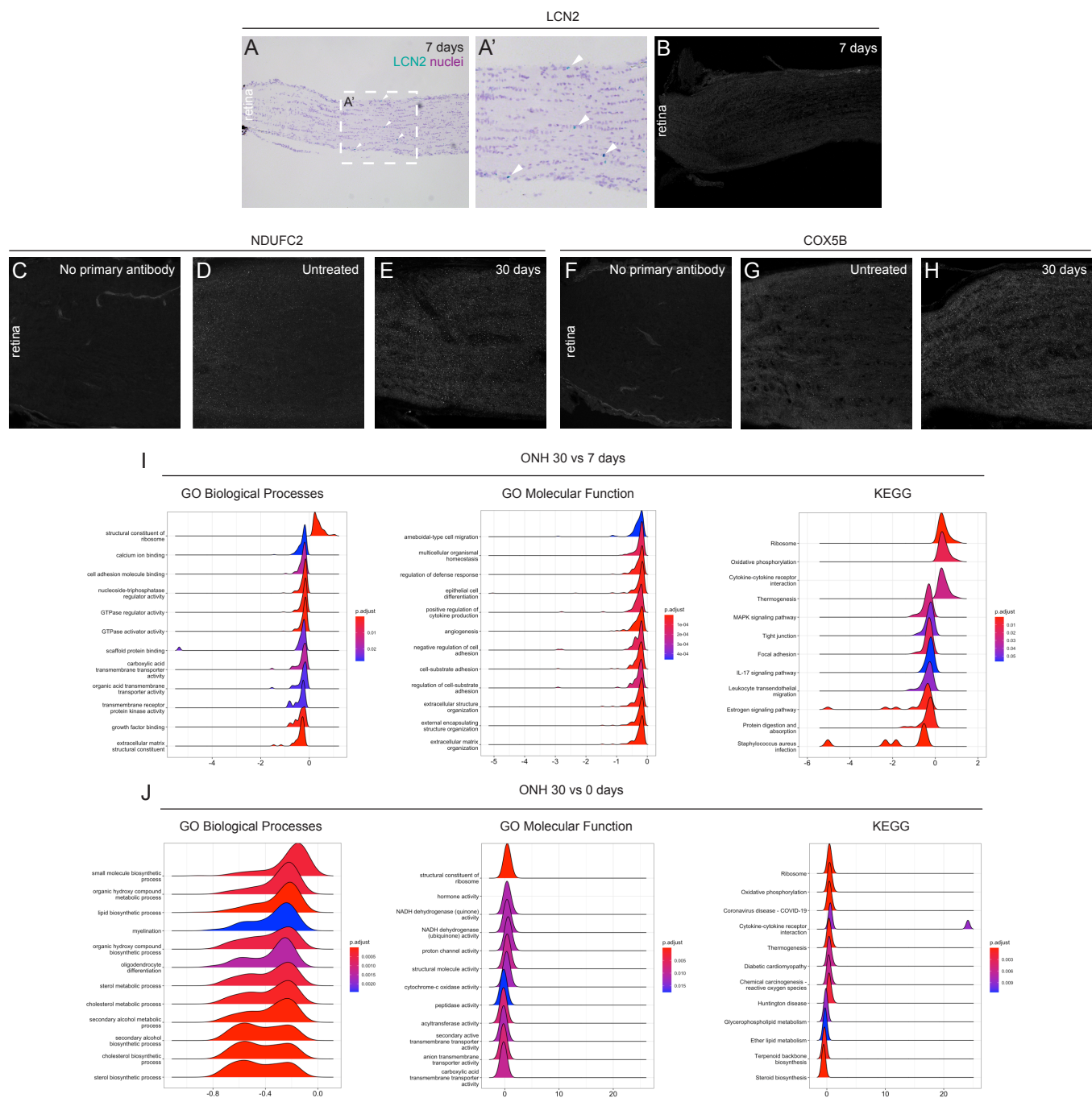

Supplementary Figure 1

Supplement: Supplementary file 1 — Additional file 1: Supplementary Figure 1. (A-B) In-situ hybridization (A, A’) and immunohistochemical staining (B) of the mouse optic nerve for LCN2. We observed very sparse labeling of LCN2 at 7 days after microbead injections (arrowheads). (C-H) Longitudinal sections of the mouse ONH region immunostained for NDUFC2 and COX5B in untreated and microbead injected mice at 30 days. (I, J) Gene set enrichment analysis using the gene ontology (GO) and KEGG database. [file 13024_2023_658_MOESM1_ESM.pdf]

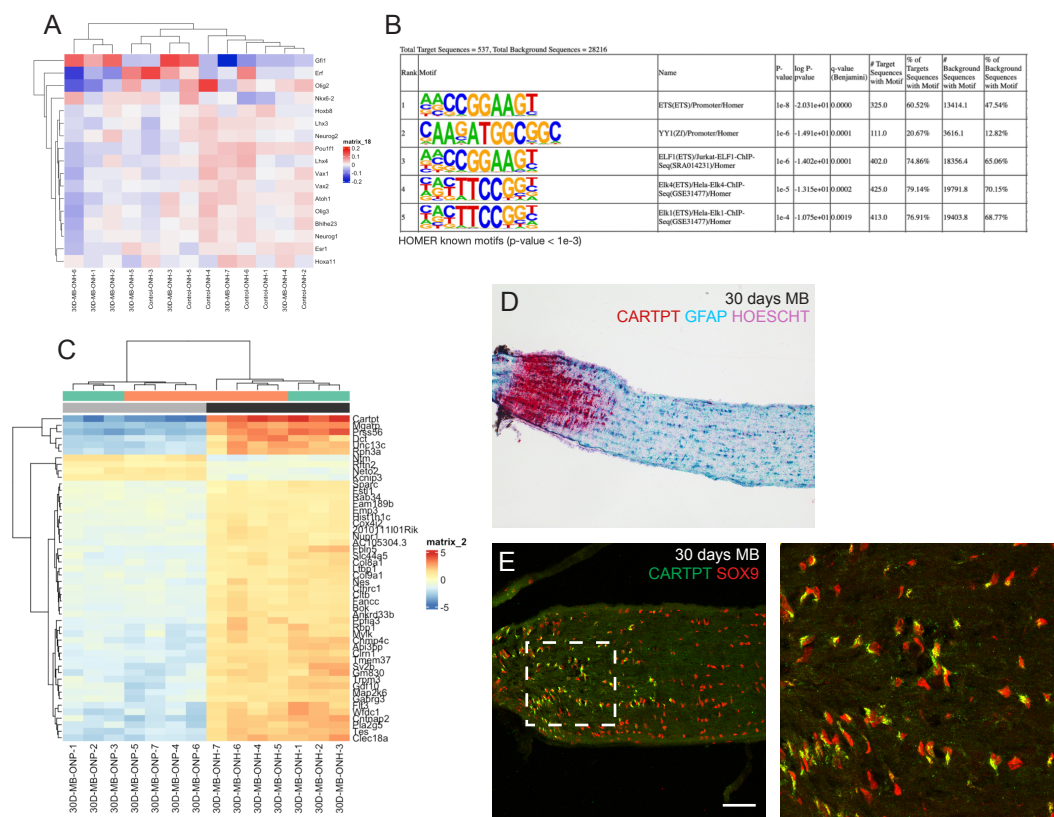

Supplementary Figure 2

Supplement: Supplementary file 2 — Additional file 2: Supplementary Figure 2. (A) Heatmap showing the per-sample activity score for each transcription factor identified as significantly different between ONH 30 vs 0 days. While there are significant differences based on the log2 fold change of genes as input, the heatmap showed limited clustering by condition. (B) Table summarizing the known motifs found to be enriched in the transcription start site +/- 2kb region of our input gene list, with a p-value < 0.001. For each known motif, the corresponding transcription factor is specified. (B) Table summarizing the known motifs found to be enriched in the transcription start site +/- 2kb region of our input gene list, with a p-value < 0.001. For each known motif, the corresponding transcription factor is specified. (D) In-situ hybridization showing the localization of Cartpt mRNA in the optic nerve of a microbead injected mouse at 30 days. (E) Longitudinal sections of the mouse ONH region showing immunostaining for CARTPT and SOX9 in microbead injected mice at 30 days. [file 13024_2023_658_MOESM2_ESM.pdf]
