# Supplementary material for: Astrocytes of the optic nerve exhibit a region-specific and temporally distinct response to elevated intraocular pressure
Source: Mol Neurodegener. 2023 Sep 27;18:68. doi: 10.1186/s13024-023-00658-9 (PMC10523752; doi:10.1186/s13024-023-00658-9)
Supplement: Supplementary file 3 — Additional file 3: Supplementary Table 1. The quantity and quality of RNA and cDNA measured using the Agilent 2100 Bioanalyzer. [file 13024_2023_658_MOESM3_ESM.docx]

|  | **Input** | | **Immunoprecipitate (IP)** | | **cDNA** | |
| --- | --- | --- | --- | --- | --- | --- |
|  | RNA yield (pg/µl) | RIN | RNA yield (pg/µl) | RIN | Input  (ng/µl) | IP  (ng/µl) |
| Optic nerve head  (pool of 4) | 253.4 ± 46.14 | 8.61 ± 0.26 | 585.5 ± 148 | 9.34 ± 0.15 | 5.91 ± 0.44 | 5.69 ± 0.65 |
| Optic nerve proper  (pool of 4) | 155 ± 78.65 | 8.36 ± 0.53 | 2284 ± 247 | 9.0 ± 0.21 | 4.8 ± 0.26 | 7.79 ± 1.91 |
